# Implementing Age-Friendly University Principles: Institutional Supports, Barriers, and Outcomes

**DOI:** 10.1093/geroni/igab046.2014

**Published:** 2021-12-17

**Authors:** Katarina Felsted, Jacqueline Eaton

**Affiliations:** 1 University of Utah, Salt Lake City, Utah, United States; 2 University of Utah, University of Utah, Utah, United States

## Abstract

The University of Utah Gerontology Interdisciplinary Program received an Age-Friendly University (AFU) seed grant through GSA’s Academy of Gerontology in Higher Education, funded by AARP, to develop a model for promoting lifelong learning in partnership with university and community stakeholders. We designed and instituted a targeting marketing campaign that supported our goals: 1) to implement AFU principles; 2) to promote awareness of HB60, a legislative bill allowing people 62+ to audit courses at public universities for a minimal cost; 3) to enhance HB 60 enrollment through increased communication of online course options and tuition waiver support; and 4) to improve university and community stakeholder engagement. This presentation describes project benefits, including increased awareness of AFU initiatives, promoting age diversity, safe participation through online coursework, and enhanced community partnerships. The initiative garnered strong departmental support for marketing, communications, and structure for the post-award process. Barriers occurred due to a lack of HB60 infrastructure at the university level, which inadvertently obstructs organizational engagement. This initiative targeted AFU principles while supporting the university’s strategic goal of engaging communities and preparing to pursue membership in the AFU Global Network. Future AFU goals include developing advocacy channels within the university to improve organizational support.

